# Can Pure Predictions of Activity Coefficients from PC-SAFT Assist Drug–Polymer Compatibility Screening?

**DOI:** 10.1021/acs.molpharmaceut.3c00124

**Published:** 2023-06-30

**Authors:** Jáchym Pavliš, Alex Mathers, Michal Fulem, Martin Klajmon

**Affiliations:** Department of Physical Chemistry, Faculty of Chemical Engineering, University of Chemistry and Technology, Prague, Technická 5, 166 28 Prague 6, Czech Republic

**Keywords:** amorphous solid dispersions, drugs, polymers, compatibility, solubility, prediction, PC-SAFT

## Abstract

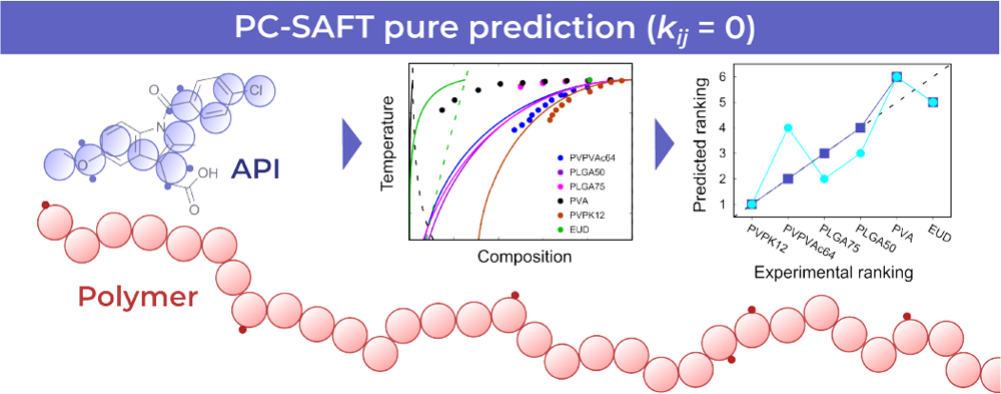

The bioavailability of poorly water-soluble active pharmaceutical ingredients (APIs) can be improved *via* the formulation of an amorphous solid dispersion (ASD), where the API is incorporated into a suitable polymeric carrier. Optimal carriers that exhibit good compatibility (i.e., solubility and miscibility) with given APIs are typically identified through experimental means, which are routinely labor- and cost-inefficient. Therefore, the perturbed-chain statistical associating fluid theory (PC-SAFT) equation of state, a popular thermodynamic model in pharmaceutical applications, is examined in terms of its performance regarding the computational pure prediction of API–polymer compatibility based on activity coefficients (API fusion properties were taken from experiments) without any binary interaction parameters fitted to API–polymer experimental data (that is, *k*_*ij*_ = 0 in all cases). This kind of prediction does not need any experimental binary information and has been underreported in the literature so far, as the routine modeling strategy used in the majority of the existing PC-SAFT applications to ASDs comprised the use of nonzero *k*_*ij*_ values. The predictive performance of PC-SAFT was systematically and thoroughly evaluated against reliable experimental data for almost 40 API–polymer combinations. We also examined the effect of different sets of PC-SAFT parameters for APIs on compatibility predictions. Quantitatively, the total average error calculated over all systems was approximately 50% in the weight fraction solubility of APIs in polymers, regardless of the specific API parametrization. The magnitude of the error for individual systems was found to vary significantly from one system to another. Interestingly, the poorest results were obtained for systems with self-associating polymers such as poly(vinyl alcohol). Such polymers can form intramolecular hydrogen bonds, which are not accounted for in the PC-SAFT variant routinely applied to ASDs (i.e., that used in this work). However, the qualitative ranking of polymers with respect to their compatibility with a given API was reasonably predicted in many cases. It was also predicted correctly that some polymers always have better compatibility with the APIs than others. Finally, possible future routes to improve the cost–performance ratio of PC-SAFT in terms of parametrization are discussed.

## 1 Introduction

Amorphous solid dispersions (ASDs) consisting of an amorphous active pharmaceutical ingredient (API) dispersed in a polymer carrier represent an efficient approach to increase the bioavailability of poorly water-soluble hydrophobic APIs.^1−3^ The optimal polymer carrier for a given API must be carefully designed, for example, with respect to its compatibility with the API under consideration. Here, compatibility is a general term that covers the solubility of a crystalline API in a polymer (described thermodynamically by solid–liquid equilibrium, SLE) and the mutual miscibility (liquid–liquid equilibrium, LLE) of an API and a polymer in the liquid/amorphous phase. The knowledge of these phase-behavior properties under various conditions in the form of phase diagrams is therefore one of the critical aspects in the rational selection of polymeric carriers.

Compatibility studies and subsequent polymer selection are routinely performed on the basis of costly and labor-intensive experiments. An alternative to the experiments is represented by computational approaches to ASD using thermodynamic tools. An established and widely used model for ASD is the Flory–Huggins (FH) theory.^4^ It is based on regular solution theory and employs the interaction parameter χ_API–poly_, which is adjusted on known API–polymer experimental data or estimated using other computational approaches. The latter includes the use of, e.g., molecular dynamics simulations or solubility parameters,^5−9^ although both are reported to have failures in estimating χ_API–poly_.^10^ Furthermore, the FH model itself and its χ parameter have both theoretical and practical limitations with respect to ASD.^10−13^ The most discussed fundamental drawback is that FH theory does not explicitly account for specific intermolecular interactions, such as hydrogen bonding. These interactions are assumed to be important in API–polymer systems. A practical drawback is, e.g., unreliable extrapolation of the solubility to room/storage temperature. We should also mention the Hansen solubility parameter (HSP) approach for qualitative compatibility screening based on the “like-dissolves-like” paradigm, which is, despite all its acknowledged drawbacks, still a popular and valuable tool for formulators.^14−16^ Other computational approaches to estimate the compatibility between APIs and high-molar-mass (high-*M*) solvents (not necessarily polymers) are also being developed and tested, e.g., on the basis of molecular simulations,^10,11,17^ quantum mechanics (QM),^18−20^ and machine learning.^21,22^ However, they appear to require deep expertise from the user.

In light of the drawbacks of the FH approach outlined above, more advanced solution theories have gained attention with respect to the modeling of ASD. The perturbed-chain statistical associating fluid theory (PC-SAFT) equation of state (EOS)^23,24^ represents one of such theories that has gained popularity in pharmaceutical applications in the past decade, including the solubility of APIs in low-*M* solvents,^25−28^ partitioning behavior of APIs (LLE),^29,30^ cocrystal screening,^31^ and phase behavior of ASD.^32−39^ PC-SAFT explicitly takes into account specific interactions, including the important hydrogen bonding, and describes materials using pure-substance parameters that have a clear physical meaning. Furthermore, it approximates molecules as chains of spherical segments, which appears to be appropriately capturing the structure of (linear) polymers, in principle. As such, PC-SAFT would meet various criteria for a model to be successful for ASD. However, it is a semiempirical and relatively highly parametrized model, which means that it uses experimental data to fit the model parameters. Specifically, PC-SAFT requires a set of five specific parameters to be known for each substance under consideration. Furthermore, for modeling mixtures, the so-called binary interaction parameter (*k*_*ij*_) is introduced into the model combining rules.^23^ This parameter allows for an *ad hoc* improvement of the calculated phase equilibria curves based on fitting to experimental mixture data. Calculations without *k*_*ij*_ (i.e., *k*_*ij*_ = 0) are usually termed “pure” or “theoretical” predictions, as they are based only on the pure substance parameters. In total, PC-SAFT needs 5 pure-component parameters per compound plus 1 *k*_*ij*_ per API–polymer mixture. For comparison, the other above-mentioned approaches to pharmaceutical systems require (in their most common application regime):

- HSP: 3 parameters per compound;
- FH: 1 parameter per compound plus 1 χ_*ij*_ per API–polymer mixture;
- machine learning: 10 and 11 molecular descriptors per API and polymer, respectively^21^ (plus large body of experimental data on API–polymer mixtures for training);
- QM-based approaches (e.g., COSMO-based ones^40,41^): 1 quantum-mechanically calculated sigma-profile per compound;
- other QM approaches that primarily focus on drug–large biomolecule intermolecular interaction typically use semiempirical parametrized but substance-independent QM methods.^20,42^

Furthermore, in the case of complex chemicals such as APIs, the performance of PC-SAFT is highly sensitive to the parametrization approach. This means that the model results for systems/properties/conditions not included in the parametrization can be unreliable and very diverse depending on the specific API parameter set used.^27,28^

It appears that numerically accurate predictions of the API solubility in normal or polymeric solvents are difficult to achieve from PC-SAFT as long as the *k*_*ij*_ between an API and a solvent is not applied.^27,28,37,39^ This is documented by the fact that almost all available studies that applied PC-SAFT to ASD did not avoid using specifically adapted *k*_*ij*_s. Therefore, PC-SAFT has been utilized more as a correlation and extrapolation tool for ASDs to date. This is not an abnormal situation, since it appears that none of the available models provides numerically accurate and reliable pure predictions of the API solubility, including even the most progressive tools.^12,28,43^ However, the question is *how much* (in)accurate the PC-SAFT pure predictions are and *whether* they can be used at least for qualitative relative estimates, consisting of ranking polymers based on the predicted solubility and miscibility values. Since the pure *k*_*ij*_-free predictions for ASD have so far been rarely reported (e.g., in ref^32^ or in our previous studies^36−39,44^), it remains to address these questions in the current study.

In this work, a set of 7 APIs, 11 polymers, and 39 API–polymer systems thereof were considered, for which reliable experimental solubility data have been measured over the past years in our laboratory. An overview and chemical identifiers of all APIs and polymers included in this work are given in Tables 1 and 2, respectively. For each API–polymer system, the SLE curve and, if present, the LLE curve were calculated by PC-SAFT with *k*_API–poly_ = 0. The data predicted by PC-SAFT in this way were then compared to their experimental counterparts both quantitatively and qualitatively. This enabled the first systematic and comprehensive evaluation of the purely predictive power of PC-SAFT for rational polymer screening in the field of ASD. Our ultimate goal is to illustrate what to expect from PC-SAFT when applied to ASD in a purely predictive regime based only on pure substance parameters, which simulates the situation in which one has to deal with a completely new API–polymer combination with no binary information available to adjust *k*_API–poly_.

**Table 1:** Overview and Chemical Identifiers of the Considered APIs

API	Abbr.	CAS RN	*M* (g mol^–1^)
Griseofulvin	GSF	126-07-8	352.77
Ibuprofen	IBP	15687-27-1	206.28
Indomethacin	IMC	53-86-1	357.79
Naproxen	NPX	22204-53-1	230.26
Nifedipin	NIF	21829-25-4	346.34
Paracetamol	PCM	103-90-2	151.16
Simvastatin	SIM	79902-63-9	418.60

**Table 2:** Overview and Chemical Identifiers of the Considered Polymers

Name^a^	Abbr.	*M* (g mol^–1^)	Monomer(s)	CAS RN
EUDRAGIT L100-55	EUD	212000	methacrylic acid (PMAA)	79-41-4
			ethyl acrylate (PEA)	140-88-5
AFFINISOL^TM^ HPMCAS 716G	HPMCAS	120000	b	71138-97-1
PDLLA	PDL	16400	lactic acid (PLLA/PDLA)	50-21-5
PLGA 50:50^c^	PLGA50	9877	lactic acid (PLLA/PDLA)	50-21-5
			glycolic acid (PGA)	79-14-1
PLGA 75:25^c^	PLGA75	12900	lactic acid (PLLA/PDLA)	50-21-5
			glycolic acid (PGA)	79-14-1
PVA (Parteck MXP)	PVA	32000	vinyl alcohol (PVA)	9002-89-5
PVP K12^c^	PVPK12	2500	*N*-vinyl-2-pyrrolidone (PVP)	88-12-0
PVP K25^c^	PVPK25	25700	*N*-vinyl-2-pyrrolidone (PVP)	88-12-0
PVP K30^c^	PVPK30	49000	*N*-vinyl-2-pyrrolidone (PVP)	88-12-0
Kollidon VA64	PVPVAc64	65000	*N*-vinyl-2-pyrrolidone (PVP)	88-12-0
			vinyl acetate (PVAc)	108-05-4
Soluplus	SOL	118000	d	402932-23-4

a Commercial/established names. Acronyms: HPMCAS = hydroxypropyl methylcellulose acetate succinate; PDLLA = poly(D,L-lactic acid); PLGA = poly(D,L-lactic-co-glycolic acid); PVA = poly(vinyl alcohol); PVP = poly(vinyl pyrrolidone).

b Hydroxypropyl methylcellulose-based backbone with several different substituents on the monomer units (see, for example, ref^45^ for further information).

c The two PLGA polymers differ in the ratio of the monomer units, while the three PVP polymers only differ in the chain length (i.e., *M*).

d Polyvinyl caprolactam-polyvinyl acetate-polyethylene glycol (see, for example, ref^46^ for further information).

This paper is set up as follows. In the next section, we provide the methodological and computational details applied in this work. The prediction results obtained from PC-SAFT EOS are then presented and thoroughly discussed in Section 3, and the findings are summarized in the last section.

## 2 Computational Methods

### 2.1 Solubility and Miscibility Calculations

The solubility of a crystalline API, that is, the equilibrium mole fraction of an API (*x*_API_) in a liquid (amorphous) API–polymer phase at a temperature *T* and pressure *p*, is given by the following SLE equation.^47^
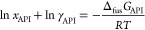
1

In eq 1, γ_API_ is the activity coefficient of an API in the solution (calculated by PC-SAFT), *R* is the universal gas constant, and Δ_fus_*G*_API_ is the difference between the molar Gibbs energies of the standard state (i.e., the pure supercooled liquid API) and the pure crystalline API. Δ_fus_*G*_API_ can be calculated from the thermodynamic properties of the pure API using the following thermodynamically rigorous relation.
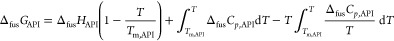
2

In eq 2, *T*_m,API_ is the melting point of the API, Δ_fus_*H*_API_ is the molar enthalpy of fusion of the API at *T*_m,API_, and Δ_fus_*C*_*p*,API_ is the difference between the isobaric heat capacity of the liquid and crystalline phases of the pure API. To calculate Δ_fus_*G*_API_ in this work, we used reliable experimental data on *T*_m,API_, Δ_fus_*H*_API_, and Δ_fus_*C*_*p*,API_. They can be found in Table 3. The activity coefficient of API (which is a function of *T*, *p*, and *x*_API_) then remains the only quantity in eq 1 calculated using the PC-SAFT EOS. In this context, the predictions of API solubility presented in this work are “pure” only in terms of γ_API_. Readers interested in methods to estimate the fusion properties are referred, e.g., to refs.^48,49^

**Table 3:** Fusion Properties of the Considered APIs

API	Form^a^	*T*_m_ (K)	Δ_fus_*H* (kJ mol^–1^)	Δ_fus_*C*_*p*_ (J K^–1^ mol^–1^)	Source^b^
GSF	I	491.85	37.90	93.84	ref^50,51^
IBP	I	348.55	26.40	176.16440 – 0.3449480 · (*T*/K)	ref^52^
IMC	γ	433.35	38.10	238.18385 – 0.2785901 · (*T*/K)	ref^52^
NPX	I	429.25	32.40	99.30	ref^52^
NIF	α	445.75	39.30	121.22	ref^50,53^
PCM	I	442.55	28.00	99.80	ref^54,55^
SIM	I	412.45	27.75	278.77100 – 0.331300 · (*T*/K)	ref^50,56^

a The exact polymorph form considered.

b If two sources are cited, the second one is for Δ_fus_*C*_p_.

The solubility curve for a phase diagram was then created from a set of *x*_API_ values calculated using eq 1 as a function of the temperature up to *T*_m,API_. To create phase diagrams and quantify the error of the PC-SAFT predictions, the resulting *x*_API_ values were recalculated to weight fractions *w*_API_, as usual in the ASD-oriented literature.

API–polymer systems can also exhibit limited miscibility, which means that they split into two coexisting amorphous phases, one with a high API concentration and one with a lower API concentration. Such an amorphous–amorphous phase separation (AAPS) is unfavorable with respect to the stability of ASD. AAPS is described thermodynamically in terms of LLE. To calculate the LLE in API–polymer systems using PC-SAFT, we used the method of alternating tangents proposed by von Solms et al.^57^ This method represents a robust and general algorithm for finding the LLE, particularly in highly asymmetric binary mixtures, such as, for example, those of a polymer and a low-*M* solvent or those of a polymer and an API. In such systems, computational difficulties may easily occur. The main advantage of the method is that it searches for the composition of only one of the equilibrium liquid phases at a time, instead of a simultaneous solution of two equilibrium points. However, the resulting binodal points must satisfy the standard equilibrium condition for LLE, i.e., the equality of the thermodynamic activities (*a*_*i*_ = *x*_*i*_ γ_*i*_) of the individual components in both liquid phases.
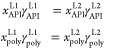
3

In eq 3, the superscripts L1 and L2 denote the two coexisting liquid (amorphous) phases, and *x*_API_^L1^ and *x*_API_^L2^ are the binodal points. A detailed description of the method can be found in the original paper.^57^ Repeating the calculation at different *T* values produces the binodal AAPS curve. Systems that are completely miscible at the studied temperature range do not show any AAPS binodal curve. In all LLE and SLE calculations performed in this study, a pressure value of 0.1 MPa was considered.

### 2.2 PC-SAFT Equation of State

The PC-SAFT EOS considers molecules to be chains of spherical segments capable of interacting with each other. The residual Helmholtz energy *a*^res^ (i.e., the difference between the Helmholtz energy of a real fluid system and the Helmholtz energy of an ideal gas) is given as a sum of individual contributions based on the properties of the molecules involved.
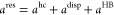
4

In eq 4, repulsive interactions are included in the hard-chain term *a*^hc^, which expresses the contribution of the size and structure of a molecule depending on the number of spherical segments (*m*) and their diameter (σ). The intermolecular attraction due to dispersion forces is described by the term *a*^disp^ and parametrized using the dispersion energy parameter (*u*/*k*_B_; *k*_B_ is the Boltzmann constant). Hydrogen bonding (HB) interactions are explicitly included using the *a*^HB^ term (often called the “association” term), which requires two additional parameters to be known: the HB energy parameter (*ε*^HB^/*k*_B_) and the effective HB volume (κ^HB^). Furthermore, the number of HB donors and HB acceptors (*N*^HB^; also known as the association scheme) must be specified. A detailed description of the contributions to *a*^res^ in eq 4 can be found in the original papers.^23,24,58,59^

The compound-specific parameters *m*, σ, *u*/*k*_B_, *ε*^HB^/*k*_B_, κ^HB^, and *N*^HB^ serve as the main input for PC-SAFT calculations. They are usually identified by fitting them to experimental data of pure substances. Liquid density and vapor pressure data are routinely used for this purpose in the case of ordinary chemicals. However, such a data set is generally inaccessible for APIs and polymers in its complete form. Therefore, the parameters for APIs are usually fitted to API solubility data in low-*M* solvents,^25,27^ while those for polymers are commonly fitted to volumetric and/or phase equilibrium data.^60^ The choice of an appropriate parametrization strategy with respect to both modeling assumptions and experimental data is not a trivial task, because it typically has a significant impact on the quality of the results obtained. For example, it has previously been shown that the choice of experimental data used in the regression of API parameters considerably affected the results for API–low-*M* solvent systems.^27,28^ Therefore, we examined two different parametrizations for some APIs to identify their effects on the compatibility predictions in ASDs (details are provided in the next section).

When applied to mixtures, such as ASD, the thermodynamic properties of a system can be theoretically predicted based only on pure-compound parameters using the standard combining rules for the cross parameters σ_*ij*_, *u*_*ij*_/*k*_B_, *ε*_*ij*_^HB^/*k*_B_, and κ_*ij*_^HB^.^23,24^ However, it is more often the case (particularly for complex systems) that the cross-dispersion energy parameter is artificially corrected to improve the agreement between calculation and experiment
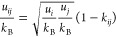
5where *k*_*ij*_ is the previously mentioned binary interaction parameter, which can be determined for each individual pair of components *i* and *j* (*i* ≠ *j*) by fitting it to known specific experimental data. Although the approach with nonzero *k*_*ij*_ can be useful in many cases (e.g., modeling/correlation and extrapolation of existing solubility data), in this work we exclusively focus on pure predictions based only on pure-compound parameters, i.e., eq 5 with *k*_*ij*_ = *k*_API–poly_ = 0 in all cases.

The activity coefficient used in both the SLE and LLE equation (eqs 1 and 3, respectively) is calculated using the following relation
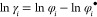
6where φ_*i*_ and φ_*i*_^•^ denote the fugacity coefficient of the component *i* in the API–polymer amorphous (liquid) mixture and in the pure liquid state, respectively. Both of them are calculated from PC-SAFT using the relation^25^
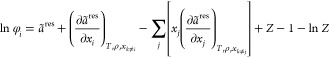
7where *ã*^res^ = *a*^res^/(*RT*) is the reduced residual Helmholtz energy (*a*^res^ is calculated using eq 4), ρ is the density, and *Z* is the compressibility factor. All calculations with PC-SAFT were performed using our own in-house codes written in Fortran.

### 2.3 PC-SAFT Parametrization Applied

The PC-SAFT parameter sets for all polymers considered in this work were taken from the literature and are provided in Table S1 in the Supporting Information. Some of them, namely, EUD, PLGAs, and PVPVAc64, were explicitly modeled using the copolymer approach within PC-SAFT formulated by Gross et al.^59^ This means that the different monomer units of a copolymer were described by their own sets of PC-SAFT parameters corresponding to a homopolymer. For example, EUD was modeled as a combination of PMAA and PEA homopolymers. In general, the parameter sets for the vast majority of the polymers were obtained by the standard parametrization approach with respect to polymers, i.e., by fitting them to experimental volumetric data of the given polymer and/or phase equilibrium (vapor–liquid or liquid–liquid) data on binary mixtures of the polymer and a low-*M* solvent. While all APIs considered in this work are modeled as self-associating (i.e., with the HB donor and HB acceptor sites and with nonzero *ε*^HB^/*k*_B_), the PC-SAFT models used for the considered polymers qualitatively differ in their association behavior, as can be deduced from the values of *ε*^HB^/*k*_B_ and *N*^HB^ shown in Table S1. PVP, PVPVAc, and SOL are considered nonself-associating (*ε*^HB^/*k*_B_ = 0), but they can form induced cross-associations^61^ with APIs because they have association sites (i.e., *N*^HB^ > 0). This approach appears to be correct with respect to their molecular structure, as they contain lone electron pairs on the oxygen atoms that can act as HB acceptor sites. Although PDL and PLGA should be modeled in the same way for the same reason, they are routinely considered completely nonassociating in PC-SAFT studies,^62,63^ which was also followed in this work. EUD, HPMCAS, and PVA are correctly considered to be self-associating.

As mentioned above, we used two different parameter sets for most of the APIs to explore the sensitivity of API parametrization. These parametrizations differed mainly in the experimental data types included in their regression. The first set for each API is termed the “reference” set (hereafter denoted REF) and represents the routine parametrization strategy for APIs based on fitting the API parameters to experimental solubility data in one or multiple pure low-*M* solvents. For consistency, all REF parameters used were those developed by a single research group.^25,32,53,64,65^ However, such reference parameters were not available for SIM. Therefore, we developed a new set for SIM, following the REF parametrization strategy represented by fitting the parameters to the experimental SIM solubility data in pure solvents, specifically alcohols^66^ (the PC-SAFT parameters for alcohols were taken from the literature^24,67^). The simulated annealing optimization technique^27,68^ was used to determine the parameters. It allows for an extensive exploration of the parameter space and thus reduces the possibility of being trapped in the local minima of the objective function to be minimized. To reduce the number of parameters fitted, we fixed the association volume parameter, κ^HB^, to a value of 0.01, as is routinely done for APIs in the literature.^25,27^ Thus, a total of four parameters (*m*, σ, *u*/*k*_B_, and *ε*^HB^/*k*_B_) were fitted. In correspondence with its HB ability, we consider this API self-associating with the symmetric six-site association scheme [i.e., 6 (3, 3)] that comprises three HB donors and three HB acceptors. This scheme was found to be the most suitable for SIM on the basis of our preliminary test calculations. The resulting set of PC-SAFT parameters for SIM is shown in Table 4.

**Table 4:** Reference (REF) and Alternative (ALT) Sets of PC-SAFT Parameter for the APIs Included in This Study

Strategy	*m*/*M* (mol g^–1^)	σ (Å)	*u*/*k*_B_ (K)	*ε*^HB^/*k*_B_ (K)	κ^HB^	*N*^HB,^^a^	Source
GSF							
REF	0.040179	3.3720	221.26	1985.49	0.02	4 (2, 2)	ref^65^
IBP							
REF	0.012226	4.4320	374.65	879.42	0.03	4 (2, 2)	ref^25^
ALT	0.026365	4.0179	309.40	516.47	0.089457	4 (2, 2)	ref^27^
IMC							
REF	0.039920	3.5350	262.79	886.40	0.02	6 (3, 3)	ref^32^
ALT	0.022072	3.8225	374.51	1295.43	0.011350	6 (3, 3)	ref^27^
NPX							
REF	0.035200	2.9390	229.45	934.20	0.02	4 (2, 2)	ref^64^
ALT	0.019162	4.1142	470.92	1202.65	0.009524	4 (2, 2)	ref^27^
NIF							
REF	0.023470	3.5810	309.44	1221.58	0.02	4 (2, 2)	ref^53^
PCM							
REF	0.049775	3.5080	398.28	1994.23	0.01	4 (2, 2)	ref^25^
ALT	0.021406	3.9819	432.09	1635.92	0.054320	4 (2, 2)	ref^27^
SIM							
REF	0.010695	3.0711	296.07	1449.58	0.01	6 (3, 3)	This work

a The numbers in the format *Z* (*X*, *Y*) are *Z*, the total number of HB (association) sites; *X*, the number of HB donor sites; and *Y*, the number of HB acceptor sites per molecule.

There are also some alternative parameter sets for APIs available in the literature. For example, we recently developed PC-SAFT parameter sets for five model APIs based not only on experimental API solubility data but also on densities and vapor pressures of pure liquid APIs^28^ (parameter sets denoted “PS4” in that work). These parameter sets were intended to improve the description of the thermodynamic reference state considered in the solubility equation (eq 1), i.e., pure (subcooled) liquid API, and were found to produce overall better results than those obtained using the routine parametrization (REF). This alternative parametrization was applied to IBP, IMC, NPX, and PCM, and was denoted “ALT”. All sets of PC-SAFT parameters for the APIs applied in this work are provided in Table 4.

### 2.4 Reference Experimental Solubility and Miscibility Data

Experimental API solubility data in polymers were used to quantify and assess the predictive power of PC-SAFT for ASDs. An overview of the API–polymer systems considered, together with the source of their respective experimental solubility data set, is provided in Table 5. In brief, all experimental data originate from our laboratory, which should ensure a desirable level of consistency in terms of the applied methodology and materials used. Specifically, they were measured by differential scanning calorimetry (DSC). From the several DSC protocols available in the literature, we prefer to use the melting point depression^69,70^ (MPD) and step-wise dissolution^37^ (S-WD) methods. The latter represents a material- and time-efficient protocol recently developed in our laboratory.

**Table 5:**
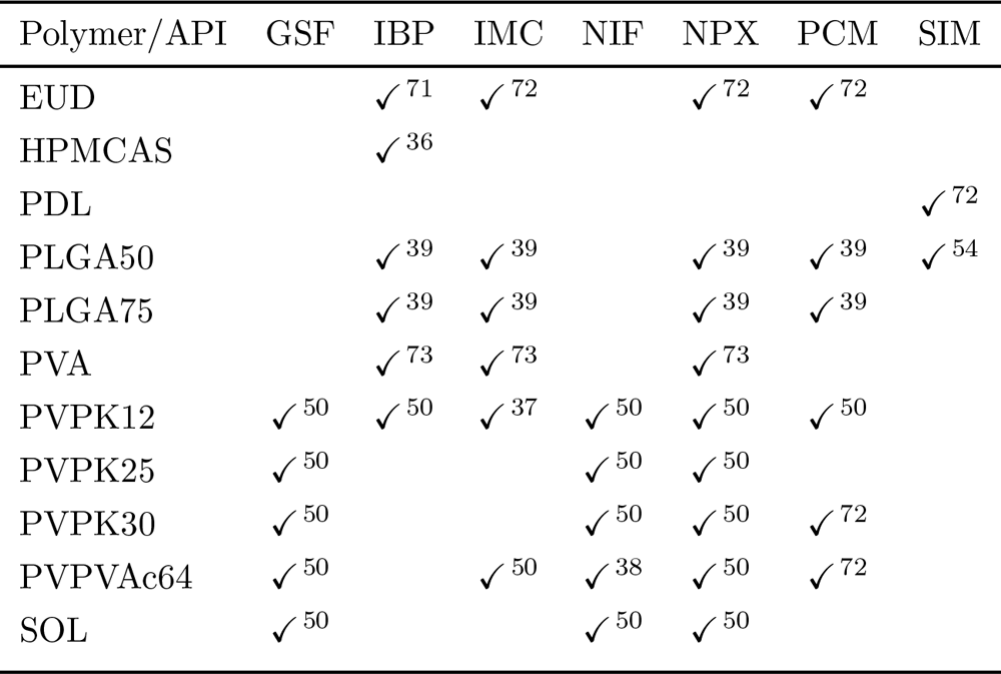
Overview of the API–Polymer Systems Considered and Sources of Their Experimental Data

The error of the API solubility predictions was primarily quantified using the average absolute relative deviation (AARD), which is calculated using the following relation.
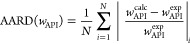
8

In eq 8, *N* is the number of experimental data points, and the superscripts “exp” and “calc” denote the experimental values and those calculated with PC-SAFT. To determine whether the model tends to over- or underestimate the experiment, we also used the average relative deviation (ARD) in addition to AARD.
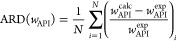
9

Furthermore, to evaluate possible correlations of predicted solubility data or their AARD or ARD values with various substance-specific descriptors, we used the coefficient of determination (*R*^2^).

With respect to miscibility (AAPS), it is generally challenging to measure in ASD, mainly due to the high viscosity of these systems.^34^ Therefore, methods different from those used for LLE in low-*M* systems must be sought. For example, AAPS can be detected during MPD measurements, where it is qualitatively manifested by the presence of two distinct glass-transition temperature (*T*_g_) values for amorphous phases rich in the API and rich in the polymer. If two *T*_g_ events are observed for an API–polymer system, then *T*_g_ is studied in more detail as a function of temperature and composition to estimate the approximate boundaries of the AAPS region.^39^ Because this approach (and any other alternative method for AAPS in ASD) is incapable of quantitatively determining the composition of the coexisting phases, the result is not a smooth binodal AAPS curve but rather a sparse net of *T*–*w*_API_ points (e.g., with a step of 10 K and 10 wt %, respectively) that qualitatively denote a single/two-phase region. For a detailed description of this methodology, see ref.^39^

## 3 Results and Discussion

This section is organized as follows. First, the quantitative performance of PC-SAFT is discussed, followed by a presentation of qualitative performance relevant to AAPS prediction and polymer ranking, for both REF and ALT parametrization of APIs.

### 3.1 Quantitative Performance

**Figure 1:**
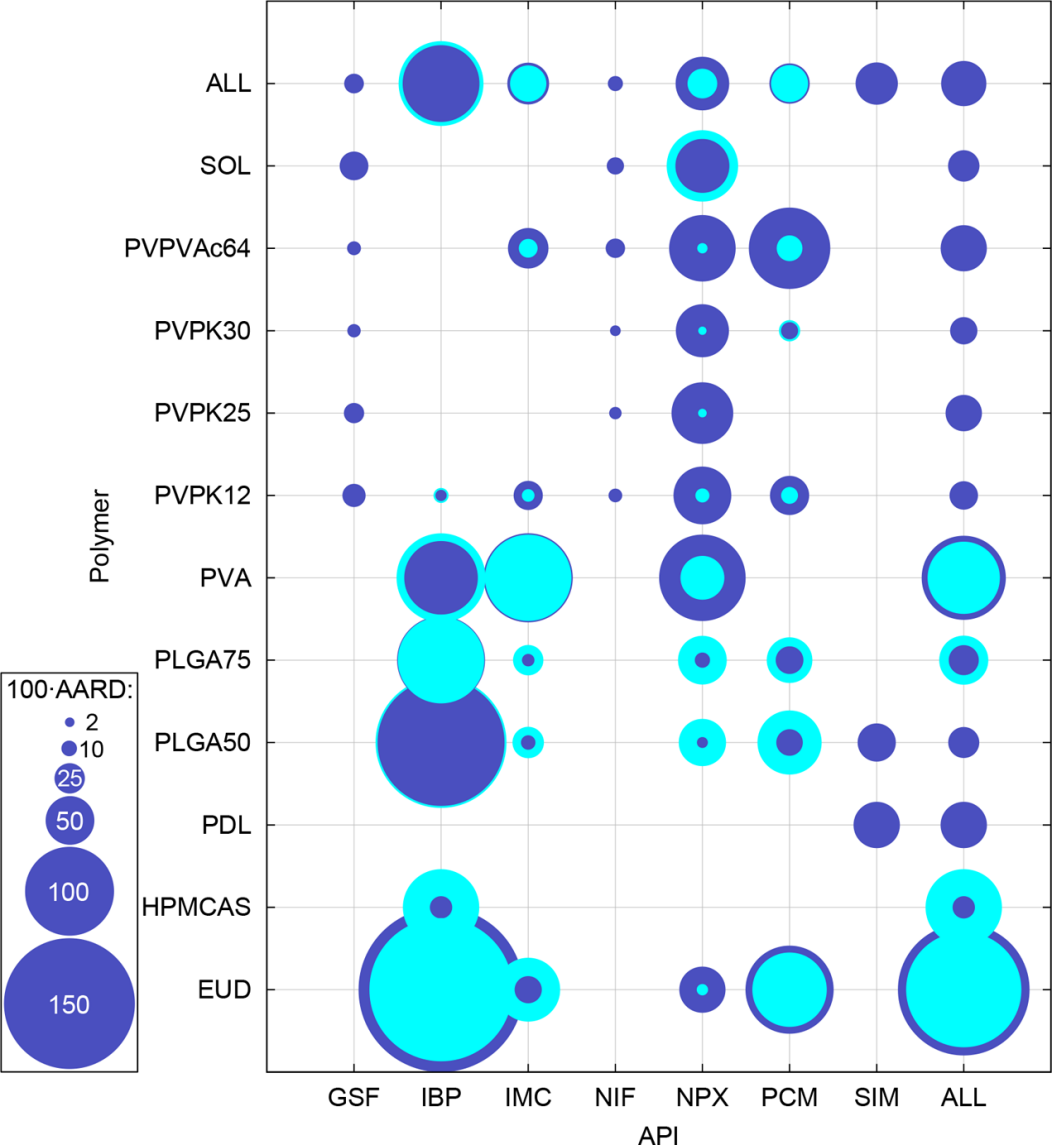
Graphical overview of the AARD(*w*_API_) values obtained from PC-SAFT with REF (dark blue) and ALT (cyan) API parametrizations for all individual API–polymer systems. Cyan circles are not shown for systems not included in the test set for ALT.

**Figure 2:**
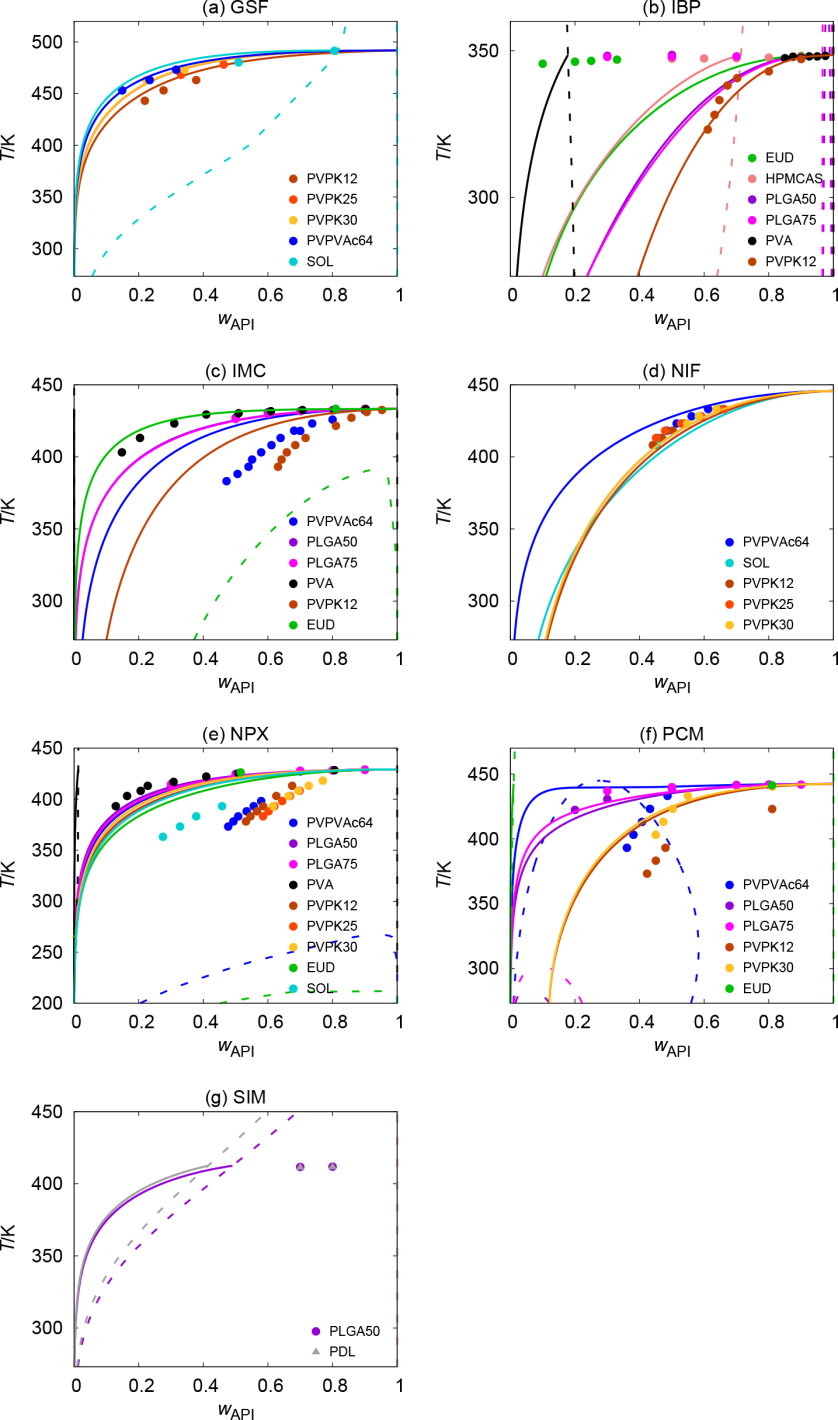
Solubility curves (solid lines) and AAPS curves (dashed lines) purely predicted by PC-SAFT with REF parametrization of APIs compared to experimental solubility data (solid circles and triangles).

**Figure 3:**
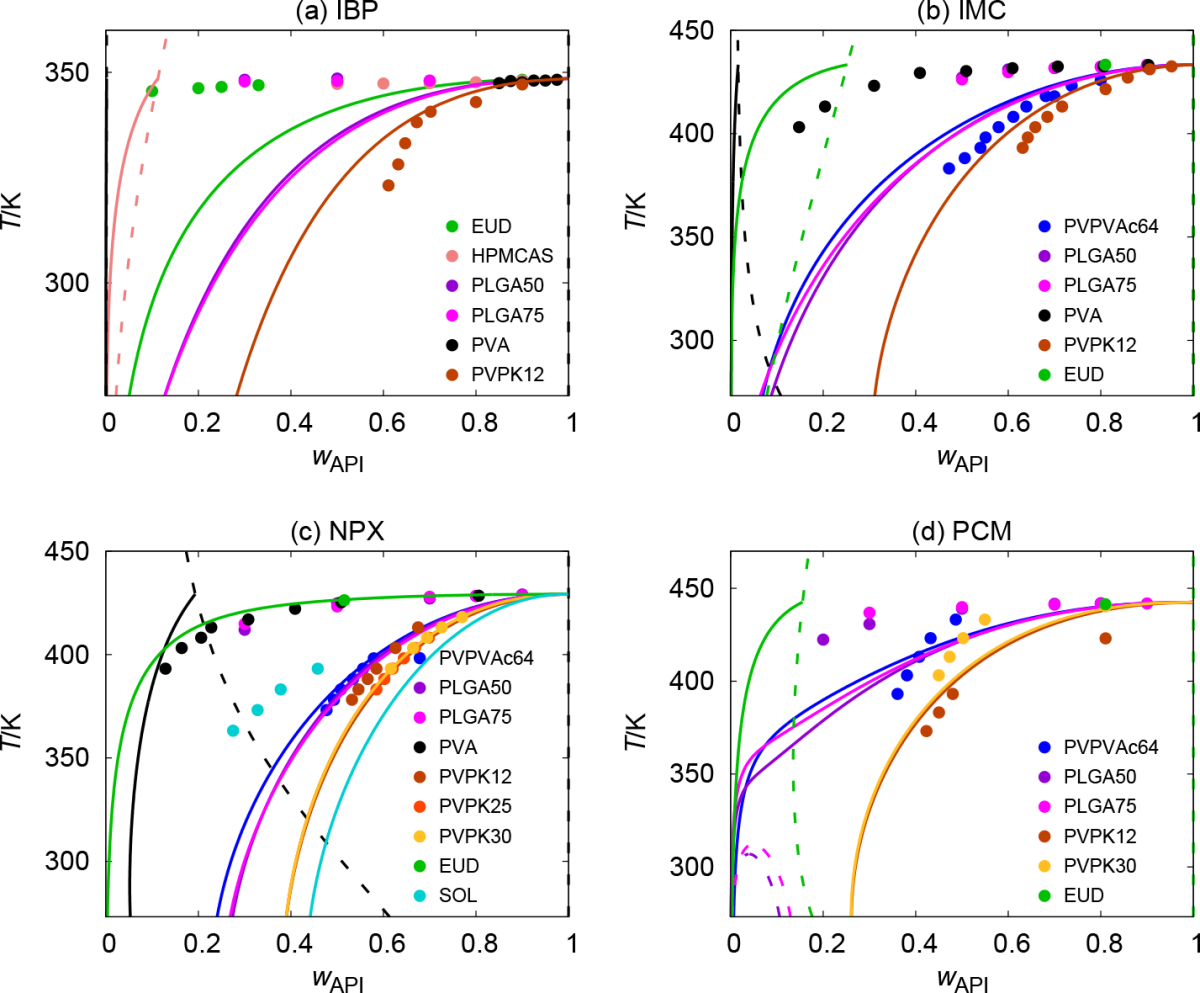
Solubility curves (solid lines) and AAPS curves (dashed lines) purely predicted by PC-SAFT with ALT parametrization of APIs compared to experimental solubility data (solid circles).

A graphical overview of the AARD(*w*_API_) values obtained from PC-SAFT for each system can be seen in Figure 1. The rows and columns represent the different polymers and APIs, respectively. The row and column named “ALL” represent the total AARD values for a given API calculated over all polymers and *vice versa*, respectively, with the upper right corner being the total AARD value calculated over all systems considered. The AARD(*w*_API_) and ARD(*w*_API_) values for individual systems are then shown in Table S2 in the Supporting Information. Phase diagrams, including solubility curves and AAPS binodal curves, compared with experimental data, are shown in Figures 2 and 3 for REF and ALT, respectively.

#### 3.1.1 REF Parametrization of APIs

The total AARD(*w*_API_) value achieved by REF for all systems was 46%. This can be considered too large for practical applications of PC-SAFT with regard to quantitative compatibility predictions. However, there are significant differences in performance with respect to individual systems (the AARD values for individual systems vary from a few percent to 200%). The prediction accuracy is promising for some systems (e.g., GSF–PVPVAc64, NIF–PVPs, and NPX–PLGAs), while it is extremely poor for some others (e.g., IBP–EUD, IBP–PLGAs, and all systems with PVA). The predominant tendency of PC-SAFT with REF is to underestimate API solubility, as indicated by the negative total ARD value calculated for all systems (−17%) and as can be deduced from Figures S1 and S2 in the Supporting Information.

Regarding individual APIs, the poorest results were obtained for IBP (total AARD of 85%). Systems of IBP with EUD, PLGAs, and PVA polymers are particularly responsible for this large total error. The reason for this high AARD may hypothetically be the fact that the set of REF parameters for IBP was trained on the solubility data only in a single solvent (acetone^25^), and such a limited chemical environment resulted in poor performance of these REF parameters for other systems. However, such a hypothesis fails when we consider that (i) the ALT parameter set for IBP, trained using a broader set of four solvents, provides even a slightly larger error (as presented in the next section), and (ii) the REF parameters for PCM, trained to a single solvent too (water^25^), show the third-best total AARD with respect to APIs. Interestingly, in our recent contribution,^27^ which examined PC-SAFT for API solubility predictions in traditional low-*M* solvents, the REF parameters for IBP led to one of the best overall results (a deviation of 0.46 log units in terms of *x*_API_), while the REF set for PCM showed a very large error of 2.3 log units. This performance is opposite to that observed in this work for API–polymer systems. In the case of IBP–PLGA combinations, it appears that the large errors (which are not seen for the other API–PLGA systems) are due to the fact that PC-SAFT, irrespective of the specific API parametrization, is unable to capture the low experimental compatibility that is enhanced even by the experimentally observed AAPS.^34,39,44^ However, for IBP–PLGA systems, PC-SAFT with REF qualitatively correctly predicted a narrow AAPS region at high *w*_IBP_ values close to unity, which would serve as a qualitative indicator of limited compatibility.

The second highest AARD value was obtained for NPX (56%). A closer inspection of Figure 1 reveals that the systems of NPX with PVP polymers are important contributors to this relatively larger AARD, although other API–PVP systems were otherwise predicted with a relatively low AARD by PC-SAFT with REF.

The best and second-best prediction accuracy was achieved for NIF and GSF, respectively, with AARDs of 9% and 14%. However, the experimental data sets for both NIF and GSF did not include systems with PVA and EUD as polymers, for which larger errors were typically observed in the case of the other APIs. Therefore, the promising predictions achieved for NIF and GSF could be the result of the absence of systems with these “problematic” polymers in the experimental test set.

Regarding individual polymers, those with high total AARD values (around 100% or even higher) are particularly EUD and PVA, as mentioned above. For PVA, a significant underestimation of API solubility is generally observed, manifested also by a large AAPS region predicted, making the APIs practically insoluble in PVA at lower temperatures. For systems with EUD, no clear trend with respect to under- or overestimation is observed. Interestingly, both PVA and EUD (together with HPMCAS) are the only self-associating polymers in the polymer selection considered in this work (see Table S1). In contrast, the lowest AARD values (20%–30%) were generally achieved for systems with PVPs, PLGAs, and SOL as polymers, i.e., those polymers considered to be nonself-associating or even completely nonassociating. While the predicted *w*_API_ values in PVPs and SOL are, in most cases, underestimated, they are overestimated in the case of PLGAs. Interestingly, this overestimation occurs despite the fact that PLGAs are, in contrast to their molecular structure, considered to be noncross-associating with APIs.^34,63^ The inclusion of association sites would then further increase the calculated solubility and, thus, the corresponding prediction error.

#### 3.1.2 ALT Parametrization of APIs

PC-SAFT with the ALT parameters for APIs produced quantitatively more accurate predictions than REF for 3 of the 4 APIs (IMC, NPX, and PCM) and for 14 of the 27 API–polymer systems that were included in the test set for ALT. At the same time, the total AARD values for REF and ALT were 55% and 45%, respectively, which means that ALT is generally slightly more numerically accurate. (Note that, for REF, the total AARD value of 55% used here is different from that presented in the previous section because it was recalculated only over IBP, IMC, NPX, and PCM for comparability with ALT.) Although the total AARD values for both REF and ALT can be considered to be more or less comparable, more significant differences are observed on the level of the individual systems. For example, for IBP–HPMCAS, the AARDs are 18% and 84% for REF and ALT, respectively, while for NPX–PVPVAc it is 72% and 3%, respectively, in favor of ALT this time. The worst results were again obtained for IBP. ALT appears to provide overall better results than REF for systems with EUD, PVPs, and PVPVAc64 as polymers, while the opposite is observed for PLGAs. Therefore, we can conclude that, with respect to individual systems, the sensitivity of the PC-SAFT results to the API parametrization is generally significant, which corresponds to what we observed earlier for pure APIs and mixtures of APIs with low-*M* solvents.^27,28^ Therefore, it is generally important to pay attention to the choice of PC-SAFT parameter sets for APIs.

Interestingly, PC-SAFT with ALT tends to predict solubility values that are generally higher than those of REF for IMC, NPX, and PCM, while the opposite is observed for IBP. This is also manifested in the fact that PC-SAFT with ALT shows, unlike REF, an overall tendency to slightly overestimate API solubility, as the total ARD value is +6%. This phenomenon can be theoretically explained by means of the differences between REF and ALT in the values of the energy parameters *u*/*k*_B_ and *ε*^HB^/*k*_B_. As can be seen in Table 4, for IMC, NPX, and PCM, *u*/*k*_B_ and *ε*^HB^/*k*_B_ values of ALT are uniformly higher than those of REF, while the opposite is true for IBP. In principle, higher values of these parameters result in stronger attractive interactions between an API and a polymer, and hence higher compatibility. This crude rule of thumb is illustrated in Figure S3a,b; it can be seen that the higher the *u*/*k*_B_ value, the higher the tendency to higher ARD(*w*_API_) values, i.e., the higher the tendency to overestimate *w*_API_ (at constant PC-SAFT parameters for polymers). For *ε*^HB^/*k*_B_, no such correlations were found. This principle can also be used to explain the fact that REF is generally better for PLGAs, while ALT is better for PVP: The REF parametrization for IMC, NPX, and PCM shows relatively low and positive errors for PLGAs, and the replacement of the REF with ALT parameter sets (which have higher values of the energy parameters) leads to higher solubilities and, thus, higher error values. Following the same principle, the initially worse performance of REF for API–PVP systems (underestimation) is improved with ALT because of the higher compatibility predicted using the latter.

PC-SAFT with ALT shows the highest error for systems with EUD, SOL, PVA, and HPMCAS as polymers. At the same time, EUD, HPMCAS, and SOL are polymers with the highest values of *M*, see Table 2. Therefore, we inspected whether there is a correlation between the prediction error and *M* of the polymers. The results are shown in Table S4a and reveal a rather slight correlation (*R*^2^ = 0.44) between AARD(*w*_API_) and polymer *M*. More information about this kind of dependence would likely be obtained by including more polymers with even higher *M* values. Similarly to our previous study,^28^ we also examined a possible correlation between the prediction error and some API descriptors, namely, *M*_API_ and *T*_m,API_ (*M*_API_ can be seen as a rough descriptor of the API molecular complexity). As shown in Figure S4b, the correlation with *M*_API_ is generally unconvincing (*R*^2^ = 0.18). However, what is interesting is that the error appears to decrease with *M*_API_ rather than the opposite, contradicting our previous results on API–low-*M* solvent systems, where the error generally increased with increasing *M*_API_. The correlation of AARD(*w*_API_) with the API melting temperature is stronger (*R*^2^ = 0.72; see Figure S4c), but again, the error decreases with *T*_m,API_, which contradicts what we obtained for API–low-*M* solvent systems. This suggests that, although there are many similarities in the PC-SAFT performance for API–polymer and API–low-*M* solvent systems (e.g., the dependence on API parametrization and quantitatively inaccurate results), there are also certain differences.

As mentioned above, the quantitatively poorest predictions from both REF and ALT were obtained, particularly for systems based on PVA and EUD. What makes these polymers different from the others considered in this work is their relatively high *M* and association behavior, as both are self-associating. Their self-associating nature means that they can form three types of hydrogen bonds: *inter*molecular polymer–polymer and API–polymer HB, and also *intra*molecular HB within polymer chains (because polymers such as PVA are large and flexible molecules).^74^ As a result, they can form strong HB-networks. For example, PVA, despite its hydrophilic and strong hydrogen bonding nature,^75−77^ has a relatively low solubility in water at room temperature. This can be explained by the strong synergistic effect of the intramolecular HB (intra-HB) and the corresponding HB networks,^74^ which can also have a significant effect on the other thermodynamic and mechanical properties of PVA.^75,78^ The solubility of self-associating APIs in PVA is also relatively low (less than 1 wt. % at room temperature).^73^ In this regard, the solubility behavior of PVA can be improved by disrupting the sequential intra-HB by a chemical modification of PVA.^79^

Since PC-SAFT can only account for the intermolecular HB,^80^ the missing description of intra-HB may be responsible for some part of the higher errors found for the systems with self-associating polymers. Although Avlund et al.^80,81^ developed a modified association term *a*^HB^ that enables the inclusion of intra-HB within the SAFT framework, its implementation and the related inevitable reparametrization of the self-associating polymers would be beyond the scope of this study.

In addition to the missing description of intra-HB, some other types of interactions are not explicitly accounted for in the model variant used in this work as well as in the previous PC-SAFT applications to ASDs (eq 4), for example, multipolar electrostatic interactions. However, we recently showed^27^ that the inclusion of the dipole moment of model APIs (IBP, IMC, NPX, and PCM) and the corresponding dipole–dipole interactions in API–solvent systems (*via* the dipolar term of Gross and Vrabec^82^) has a negligible effect on PC-SAFT performance. Furthermore, even if all relevant interactions were included in the model, it should be kept in mind that SAFT-type models use a general EOS expression derived using perturbation theories from simplified force fields, which typically only qualitatively describe the real interactions of the given substance(s).^83^ Therefore, the quantitative results of the SAFT-type models rely on parametrization.

Thus, from the results presented in this study, it cannot be clearly identified whether the large errors found for the self-associating polymers originate from their high *M*, the absence of description of some type(s) of interactions (intra-HB or others), or other effects. The parametrization approach can also be of great importance in the case of polymers. However, it appears that PVA and EUD were parametrized similarly to the other polymers, in general (see Table S1). Either way, a reparameterization using a different/broader experimental data set (while keeping the mathematical form of the model as is) could improve the PC-SAFT performance for systems with PVA and EUD.

Finally, it can be seen in Figures 2 and 3 that, in many cases, the purely-predicted solubility curves do not correctly capture the temperature dependence of their experimental counterparts. For example, this applies to the IBP–HPMCAS system calculated with REF and the PCM–PVPVAc64 system calculated with ALT. Although the resulting *T*-dependence is, in principle, a combination of *T*-dependencies of both γ_API_ and Δ_fus_*G*_API_ in eq 1, the incorrect *T*-dependencies of *w*_API_ may be, to a large degree, attributed to γ_API_, since we used the thermodynamically rigorous relation for Δ_fus_*G*_API_ including Δ_fus_*C*_*p*,API_ (see eq 2) to eliminate the errors that could originate from the experiment-based Δ_fus_*G*_API_ term.^27^ In such cases, the adjustment of *T*-independent *k*_API–poly_ improves the numerical agreement between the calculated and experimental values of *w*_API_, but it does not necessarily fix the incorrect *T*-dependence. Therefore, for quantitative modeling, correlation, and extrapolation, (linearly) *T*-dependent *k*_API–poly_ are sometimes applied in literature studies (e.g., refs^53,63,64^). In addition of the modeling of ternary systems (e.g., API–polymer–water^33,84,85^) based on binary information, one of the main goals of the ASD modeling with PC-SAFT so far was to extrapolate the binary API–polymer experimental data, typically measured at higher temperatures not far from *T*_m,API_, to lower *T* corresponding to the storage temperature (298 K). Therefore, long-range extrapolations with linearly *T*-dependent *k*_API–poly_ functions trained to temperatures around *T*_m,API_ (which can be as high as 490 K; see Table 3) to 298 K can be unsafe, in terms of numerical values of *w*_API_.

### 3.2 Qualitative Performance

In this section, we focus on qualitative aspects of the purely predictive performance of PC-SAFT for API–polymer systems. Specifically, we evaluated the ability of PC-SAFT to qualitatively predict AAPS and correctly order the polymers, with respect to their compatibility with the APIs.

#### 3.2.1 Prediction of AAPS

In addition to the solubility of a crystalline API in a polymer, AAPS is also an important factor with respect to API–polymer compatibility. Therefore, we also computationally screened all systems for the potential AAPS purely predicted by PC-SAFT. AAPS was studied in a temperature range from 200 to 600 K. It can be seen in Figures 2 and 3 that PC-SAFT predicts AAPS for some systems. The main question here is whether these AAPS predictions meet reality. For a better overview, we summarize the results of the computational AAPS analysis (Table S3 in the Supporting Information and compared them with experimental observations (based on *T*_g_ measurement, as described in Section 2.4).

From an experimental perspective, the presence of an AAPS region was observed for only four of the 39 systems considered, namely, IBP–PLGA50, IBP–PLGA75, NPX–PLGA75, and NPX–PVA. The presence of an AAPS in these systems is very well-captured by PC-SAFT with REF, as it qualitatively predicted an AAPS region for three of them. We use the term “qualitatively” because the predicted AAPS curve for both IBP–PLGA50 and IBP–PLGA75 is much narrower with respect to composition than that observed experimentally.^39^ PC-SAFT with ALT predicted an AAPS only for one of them (NPX–PVA). There are also a few systems for which an experimental AAPS analysis has not been performed (4 systems). PC-SAFT tends to predict AAPS for all of them, but it cannot be determined whether it is a correct prediction or not. PC-SAFT with REF also predicts an AAPS for nine other systems for which the presence of an AAPS has not been experimentally observed, e.g., PCM–PLGAs.^39^ For the remaining 22 systems (i.e., the majority), PC-SAFT correctly predicts the absence of an AAPS. This applies, for example, to all NIF-based systems or to all systems with PVP as polymers.

There are also some general observations regarding the AAPS predictions. First, it can be seen in Table S3 that if an AAPS is predicted with the ALT parametrization, then REF predicts an AAPS too. This can be again explained using the values of the energy parameters, as done in Section 3.1.2: Since those of REF are generally lower, they lead to weaker mutual API–polymer attractions and, thus, a lower solubility/miscibility. If an AAPS is predicted with ALT, then it hardly vanishes when switching to REF. Next, PC-SAFT shows a general tendency to predict AAPS, particularly for systems with IBP and PCM as APIs, and for EUD, PLGAs, and PVA as polymers. Furthermore, for PVA, large AAPS regions with respect to both temperature and composition are typically predicted.

#### 3.2.2 Polymer Ranking

**Figure 4:**
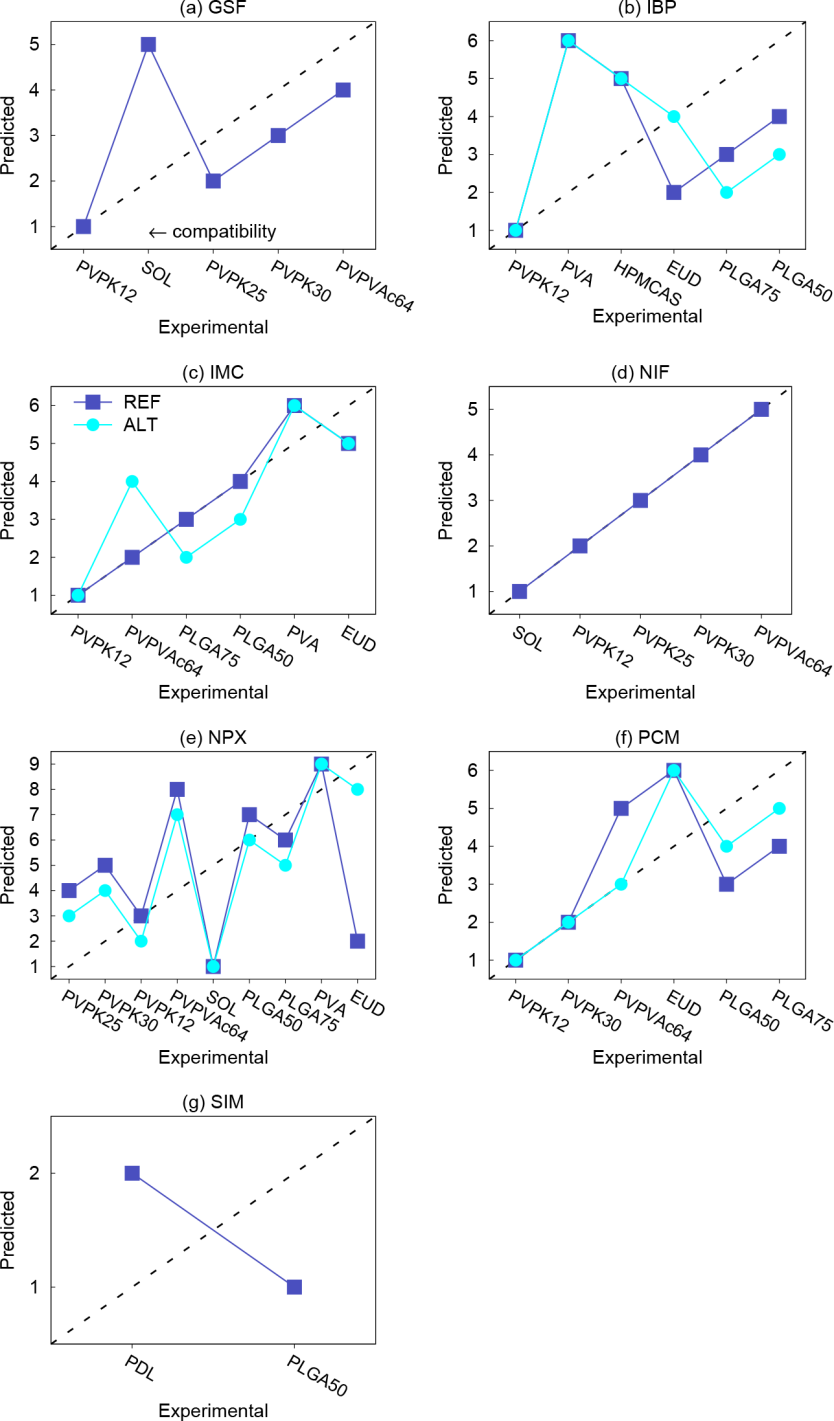
Ranking of the polymers with respect to their compatibility with the APIs predicted by PC-SAFT with the two API parametrizations compared to the experimental polymer ranking. For each API, the compatibility increases from right to left. The lines are to guide the eye.

We illustrated in Section 3.1 that the overall numerical accuracy of the pure predictions from PC-SAFT is limited. However, if we ignore the numerical (dis)agreement between the calculated and experimental values and focus only on the predicted order of polymers with respect to the solubility values at a certain temperature point or range, then we can assess the qualitative performance of a model for polymer screening. This is what we did in this work and present in this section. For each API, we ordered the polymers with respect to the predicted compatibility of them with the API and compared the order to that from the experiment. The polymer order for each API was determined on the basis of the predicted solubility (*w*_API_) values at the lowest possible experimental temperature that was common to all systems of the given API. (In the case of systems with only one experimental data point, typically close to *T*_m,API_, we adjusted a temperature-independent *k*_API–poly_ to this point and then used PC-SAFT with the obtained *k*_API–poly_ to extrapolate the experimental data to the desired temperature.) Furthermore, if PC-SAFT qualitatively predicted an AAPS for a system, the corresponding polymer automatically obtained a penalty, which means that it has been moved one position down in the compatibility ranking. The resulting polymer ranking for all APIs is shown in Figure 4 by means of diagonal graphs. The *x*- and *y*-axes represent the experimental and predicted ranking, respectively, in the order from the best compatible polymer to the worst (1st, second, third, etc.).

We first focus again on the APIs that were only covered by REF, i.e., GSF, NIF, and SIM. For GSF, all polymers are ordered correctly except SOL, for which PC-SAFT incorrectly underestimates the compatibility with GSF, also with an AAPS region (Figure 2a). For NIF, not only were the quantitative results very good but also the qualitative polymer ranking, as the polymer order predicted by PC-SAFT with REF perfectly agrees with the experiment. This is not the case for SIM; however, despite the incorrect ordering of PLGA50 and PDL, PC-SAFT correctly predicts that the compatibility of both polymers with SIM is very similar.

With regard to the remaining APIs, it can be seen that the polymer order predicted by both REF and ALT is similar, except for a few differences. For IBP, both REF and ALT incorrectly predicted HPMCAS and PVA to be the least compatible polymers, which somewhat spoils the overall diagonal graph for IBP. However, PVPK12 was correctly predicted to be the most compatible with IBP. For IMC, the predicted polymer rating is not perfect but can be considered satisfying; both REF and ALT correctly found PVA and EUD to have the worst compatibility, despite the fact that their mutual order is predicted incorrectly. Moreover, the order of the remaining polymers predicted by REF for IMC matches that of the experiment. PVPK12 is again identified as the best compatible polymer. Both REF and ALT incorrectly predicted that NPX had the best compatibility with SOL. Otherwise, the predicted polymer order would seem to be reasonable except for the failure of REF for NPX–EUD. For PCM, the order obtained from both REF and ALT is reasonable except for PCM–EUD.

With regard to the polymers, both REF and ALT correctly predicted the PVP polymers to have generally better compatibility with the APIs than the other polymer types. For example, it was always correctly identified that the PVP polymers had generally better compatibility than PVPVAc64, PLGA, or PVA and that PVPVAc64 had better compatibility than PVA. Furthermore, PVA was correctly predicted to generally have poor compatibility with the APIs, despite the poor quantitative performance (large AARDs) for PVA. On the other hand, the heavy outliers in the diagonal graphs in Figure 4 are most often those of SOL, EUD, and PVPVAc64. At the same time, SOL and EUD were also among those polymers for which PC-SAFT showed the poorest quantitative accuracy, as discussed in Section 3.1. This is another indication that a closer look should be taken in the future into the modeling (particularly with respect to the HB behavior) and parametrization approach for these “problematic” self-associating polymers.

## 4 Conclusion

The existence of a reliable computational framework for predicting the API–polymer compatibility would substantially decrease material, labor, and time consumption at the drug formulation stage. In this work, we systematically examined the PC-SAFT EOS for its potential to play this predictive role, without any fitting to specific API–polymer experimental data (i.e., without fitted *k*_*ij*_s).

We showed that the quantitative accuracy of the solubility predictions varies significantly from system to system. For a given system, the accuracy also significantly varies with the API parametrization, which corresponds to our previous findings on API–low-*M* solvent systems and to the general narrative about the high sensitivity of the PC-SAFT results to the parametrization of complex compounds, such as APIs. The highest errors are found for IBP-based systems and for systems with self-associating polymers EUD and PVA. When the results are averaged over a sufficiently large test set of API–polymer systems (as in this work), the average error is around 50% for both API parametrizations considered. Therefore, an additional treatment is necessary to rationalize the numerical accuracy of PC-SAFT. A conservative treatment is the adjustment of *k*_*ij*_s to known experimental API–polymer data. Other possible/future approaches are represented by the application of progressive methods to estimate *k*_*ij*_ based on, e.g., machine learning.^86^ However, these generally require a sufficiently large training set of experimental data to work reasonably well.

However, the PC-SAFT predictions showed a nonnegligible potential to qualitatively rate the polymers with respect to their compatibility with a given API. For example, it could correctly or at least satisfactorily predict the polymer order for GSF, IMC, and NIF, and that some polymers always have better compatibility with the APIs than others (e.g., that PVPs are better than PLGAs, or that PVPVAc64 is better than PVA). However, in other cases, the qualitative predictions were less successful (e.g., for IBP and polymers EUD and SOL), which indicates that this capability is not absolute; however, none of the available models appears to be absolutely reliable for screening purposes.^28^

For a model to be successful in ASD design, not only is the quantitative/qualitative performance critical but also the parametrization costs and the resulting cost–performance ratio. For PC-SAFT, an unfavorable aspect in this regard is the notoriously challenging and relatively expensive parametrization of both APIs and polymers. (On the other hand and in contrast to, e.g., the FH model, PC-SAFT, as an EOS, provides not only activity coefficients but also other thermodynamic properties of the studied mixture as well as of the individual components in their pure fluid state.) However, the model could be coupled with a progressive framework to overcome the parametrization drawbacks. As recently discussed by Kuentz and Bergström,^87^ it seems more logical to build a predictive tool for ASDs based not on a single model/theory but on a synergistic combination of more different approaches. Following these general ideas, PC-SAFT in conjunction with, e.g., the mentioned machine learning^86,88,89^ or QM^90^ approaches to estimate the model parameters would represent a possible coupling that should be explored in future to see whether it may keep or even improve the predictions, with the favorable reduction of the parametrization costs at the same time.

## Supporting Information Available

- PC-SAFT parameter sets for the polymers
- AARD(*w*_API_) and ARD(*w*_API_) values for API solubilities purely predicted by PC-SAFT
- Correlation of AARD(*w*_API_) or ARD(*w*_API_) values with various molecular descriptors
- Results of the computational analysis of AAPS purely predicted by PC-SAFT (PDF)
