# Supplementary material for: Can Pure Predictions of Activity Coefficients from PC-SAFT Assist Drug–Polymer Compatibility Screening?
Source: Mol Pharm. 2023 Jun 30;20(8):3960–74. doi: 10.1021/acs.molpharmaceut.3c00124 (PMC10410664; doi:10.1021/acs.molpharmaceut.3c00124)
Supplement: Supplementary file 1 — mp3c00124_si_001.pdf [file mp3c00124_si_001.pdf]

Supporting Information

Can Pure Predictions of Activity Coefficients from PC-SAFT Assist Drug–Polymer Compatibility Screening?

Jáchym Pavliš, Alex Mathers, Michal Fulem, and Martin Klajmon*

Department of Physical Chemistry, Faculty of Chemical Engineering, University of Chemistry and Technology, Prague, Technická 5, 166 28 Prague 6, Czech Republic

E-mail: martin.klajmon@vscht.cz

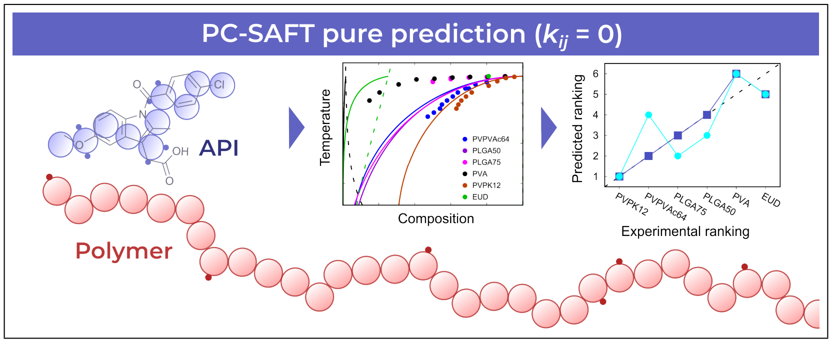

Table S1: PC-SAFT Parameter Sets for the Polymers Included in This Study

Polymer	Unit	m/M (mol g^{-1})	σ (Å)	u/k_B (K)	ε^{HB}/k_B (K)	κ^{HB} –	$N^{HB,a}$ –	w^b –	Source
EUD	PMAA[c]	0.02400	3.7000	249.50	2610.00	0.07189	2280 (1140, 1140)	0.462	[1]
	PEA[c]	0.02710	3.6500	229.00	0	0	0	0.538	[2]
HPMCAS	[d]	0.04890	2.8890	298.05	1602.30	0.02	1490 (745, 745)	1	[3]
PDL	PDLLA[e]	0.03699	3.1200	240.00	0	0	0	1	[4]
PLGA50	PLLA[e]	0.04545	2.9200	230.00	0	0	0	0.277	[4]
	PDLLA[e]	0.03699	3.1200	240.00	0	0	0	0.277	[4]
	PGA[f]	0.03130	2.8600	233.90	0	0	0	0.446	[5–7]
PLGA75	PLLA[e]	0.04545	2.9200	230.00	0	0	0	0.394	[4]
	PDLLA[e]	0.03699	3.1200	240.00	0	0	0	0.394	[4]
	PGA[f]	0.03130	2.8600	233.90	0	0	0	0.212	[5–7]
PVA	PVA[c]	0.03570	3.2993	302.20	2808.15	0.02511	1454 (727, 727)	1	[8]
PVPK12	PVP[g]	0.04070	2.7100	205.60	0	0.02	44 (22, 22)	1	[9]
PVPK25	PVP[g]	0.04070	2.7100	205.60	0	0.02	462 (231, 231)	1	[9]
PVPK30	PVP[g]	0.04070	2.7100	205.60	0	0.02	880 (440, 440)	1	[9]
PVPVAc64	PVP[g]	0.04070	2.7100	205.60	0	0.02	700 (350, 350)	0.6	[9]
	PVAc[c]	0.03211	3.3972	204.65	0	0.02	606 (303, 303)	0.4	[10]
SOL		0.05400	2.8090	225.00	0	0.02	4972 (2486, 2486)	1	[11]

[a] The number of association sites corresponds to the chain lengths (*i.e.*, M) of the polymer molecules considered in this work.
[b] Weight fraction of a given monomer unit in the polymer molecule.
[c] Parameterized to experimental volumetric data of pure homopolymer and phase equilibrium data of a binary homopolymer–low-M_w solvent system.
[d] Parametrized to experimental volumetric and phase equilibrium data of binary polymer–low-M_w solvent systems.
[e] Parameterized to experimental volumetric data of pure homopolymer.
[f] Estimated using a group contribution approach.
[g] Parametrized to experimental phase equilibrium data of a binary homopolymer–low-M_w solvent system.

Table S2: Overview of AARD(w_{API}) and ARD(w_{API}) Values Obtained in This Work for API Solubilities Purely Predicted by PC-SAFT with Two Different API Parametrizations

System	REF		ALT	
	100·AARD	100·ARD	100·AARD	100·ARD
GSF–PVPK12	18.6	−18.6	–	–
GSF–PVPK25	15.0	−15.0	–	–
GSF–PVPK30	6.5	2.0	–	–
GSF–PVPVAc64	7.1	−6.2	–	–
GSF–SOL	25.4	−25.4	–	–
GSF–ALL	14.2	−12.6	–	–
IBP–EUD	194	193.0	167	166.0
IBP–HPMCAS	17.5	1.6	84.2	−84.2
IBP–PLGA50	147	147	152	152
IBP–PLGA75	98.6	98.6	96.8	96.8
IBP–PVA	81	−80.8	100	−99.8
IBP–PVPK12	3.8	2.3	8.5	−8.5
IBP–ALL	84.8	49.4	94.7	22.5
IMC–EUD	23.5	23.5	68.8	−68.8
IMC–PLGA50	7.8	7.8	28.8	28.8
IMC–PLGA75	5.4	5.2	27.5	27.5
IMC–PVA	100	−100	96.6	−96.6
IMC–PVPK12	26.0	−26.0	5.6	−5.6
IMC–PVPVAc64	40	−40.0	13.1	−13.1
IMC–ALL	41.6	−37.2	34.8	−21.1
NIF–PVPK12	6.9	6.9	–	–
NIF–PVPK25	5.3	5.3	–	–
NIF–PVPK30	3.2	3.2	–	–
NIF–PVPVAc64	13.9	−13.9	–	–
NIF–SOL	11.3	11.3	–	–
NIF–ALL	8.6	3.0	–	–
NPX–EUD	47.0	47.0	4.0	−4.0
NPX–PLGA50	3.5	2.2	48.3	48.3
NPX–PLGA75	8.7	5.5	50.1	50.1
NPX–PVA	96.8	−96.8	44.1	−44.1
NPX–PVPK12	61.3	−61.3	7.3	7.3
NPX–PVPK25	66.1	−66.1	0.6	−0.6
NPX–PVPK30	55.5	−55.5	0.2	0.2

(Continued on next page)

Table S2: Overview of AARD(w_{API}) and ARD(w_{API}) Values Obtained in This Work for Solubilities Purely Predicted by PC-SAFT with Two Different API Parametrizations *(Continued)*

System	REF		ALT	
	100·AARD	100·ARD	100·AARD	100·ARD
NPX–PVPVAc64	72.1	−72.1	2.7	−2.7
NPX–SOL	56.6	−56.6	78.1	78.1
NPX–ALL	56.2	−52.0	26.7	10.3
PCM–EUD	98.8	−98.8	81.8	−81.8
PCM–PLGA50	22.7	22.7	69.2	69.2
PCM–PLGA75	24.1	20.5	46.3	45.9
PCM–PVPK12	38.4	−38.4	10.8	−10.8
PCM–PVPK30	11.0	−7.0	16.2	16.2
PCM–PVPVAc64	90.5	−90.5	21.8	−4.0
PCM–ALL	39.8	−20.9	35.7	20.5
SIM–PDL	47.2	−47.2	–	–
SIM–PLGA50	37.1	−37.1	–	–
SIM–ALL	42.2	−42.2	–	–
ALL–EUD	153	133	132	101
ALL–HPMCAS	17.5	1.6	84.2	−84.2
ALL–PDL	47.2	−47.2	–	–
ALL–PLGA50	28.3	20.1	60.9[a]	60.9[a]
ALL–PLGA75	27.1	25.1	50.5	50.4
ALL–PVA	93.9	−93.9	79.2	−79.2
ALL–PVPK12	25.1	−21.6	7.6[a]	−4.2[a]
ALL–PVPK25	34.9	−30.8	0.6[a]	−0.6[a]
ALL–PVPK30	23.7	−19.9	8.2[a]	8.2[a]
ALL–PVPVAc64	47.0	−46.9	12.3[a]	−8.4[a]
ALL–SOL	28.7	−17.5	78.1[a]	78.1[a]
ALL–ALL	45.8	−17.5	45.0[a]	5.6[a]

[a]Note that, for PLGA50, all PVPs, PVPVAc64, and SOL, the AARD and ARD values for REF and ALT are not fully comparable, as some of the API–polymer systems were not included in the test set for ALT.

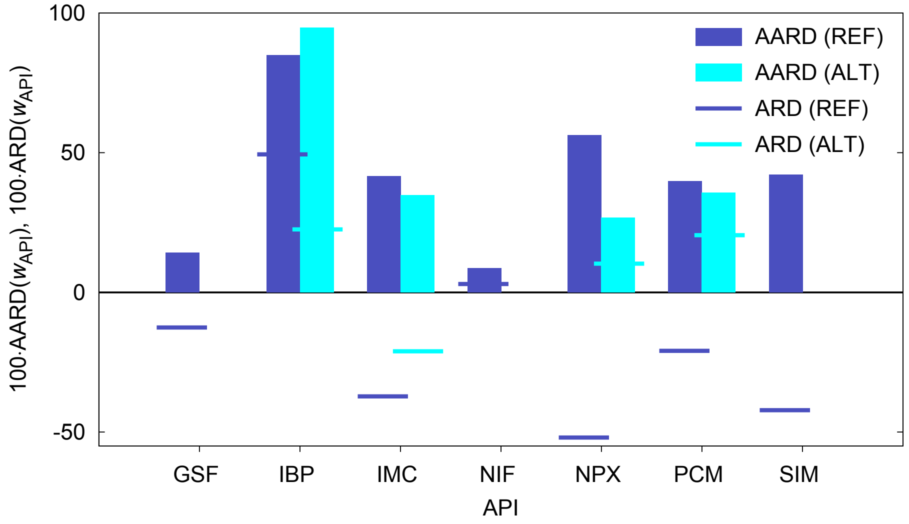

Figure S1: Overview of AARD(w_{API}) and ARD(w_{API}) values obtained in this work for API solubilities predicted by PC-SAFT for each of the considered APIs over all solvents.

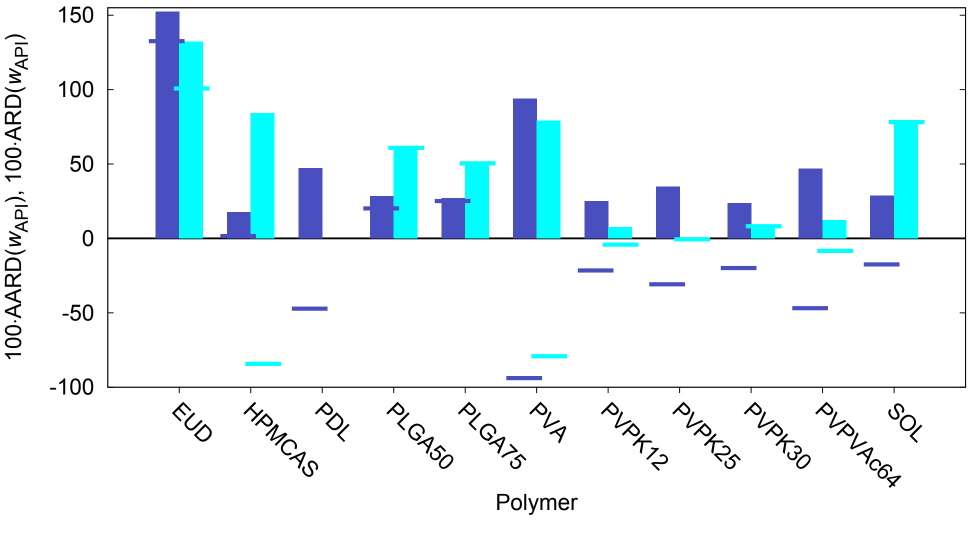

Figure S2: Overview of AARD(w_{API}) values obtained in this work for API solubilities predicted by PC-SAFT for each of the polymers over all APIs. For the legend, see Figure S1. Note that, for PLGA50, all PVPs, PVPVAc64, and SOL, the AARD and ARD values for REF and ALT are not fully comparable, as some of the API–polymer systems were not included in the test set for ALT.

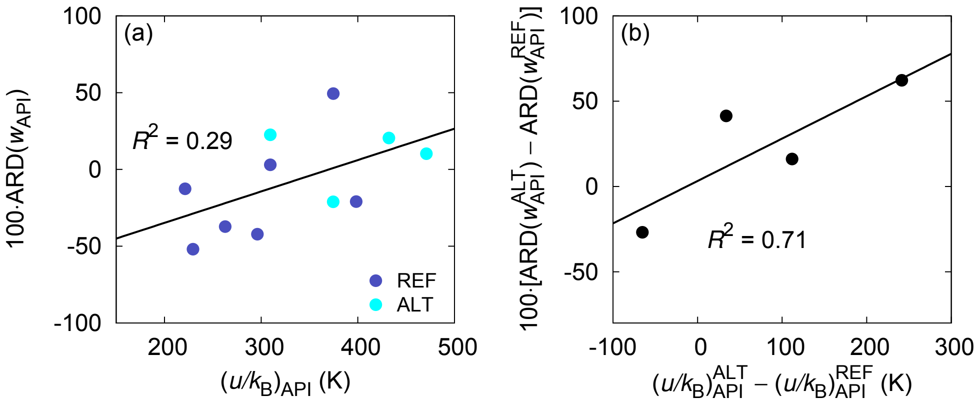

Figure S3: Correlation of ARD(w_{API}) with the API dispersion energy parameter value.

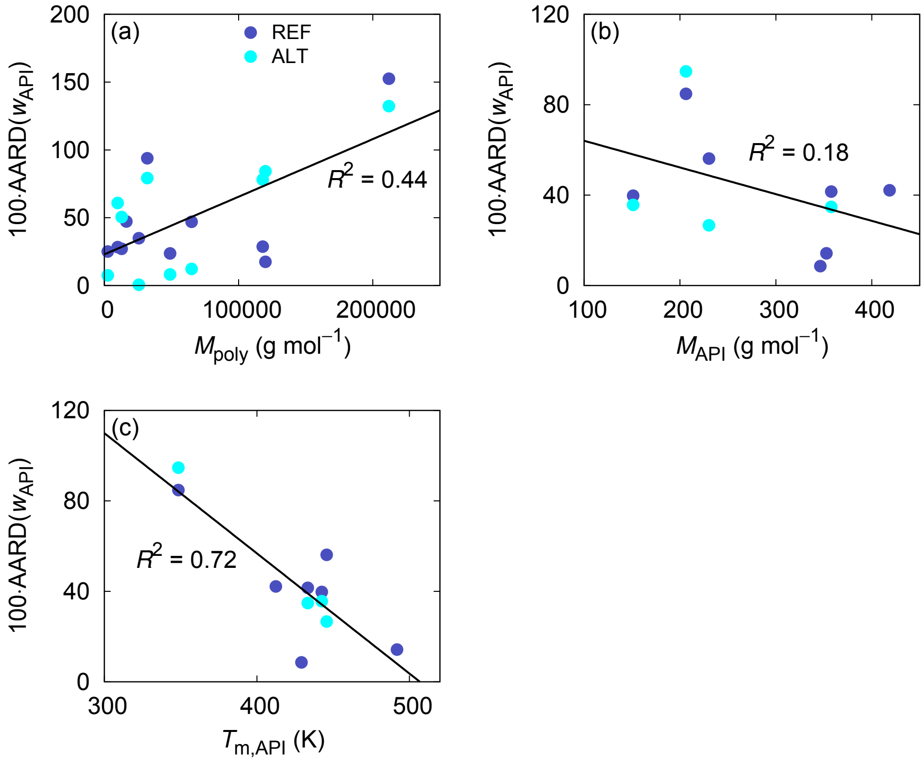

Figure S4: Correlation of AARD(w_{API}) with (a) polymer molar mass; (b) API molar mass; and (c) API melting temperature.

Table S3: Results of the Computational Analysis of AAPS Purely Predicted by PC-SAFT with Two Different API Parametrizations[a]

System	REF AAPS? (UCST)	ALT AAPS? (UCST)	Exp. evidence of AAPS
GSF–PVPK12	×	–	×[12]
GSF–PVPK25	×	–	×[12]
GSF–PVPK30	×	–	×[12]
GSF–PVPVAc64	×	–	×[12]
GSF–SOL	✓ (≫ 600 K)	–	×[12]
IBP–EUD	×	×	×[13]
IBP–HPMCAS	✓ (≫ 600 K)	✓ (≫ 600 K)	×[14]
IBP–PLGA50	✓ (≈ 375 K)	×	✓[15]
IBP–PLGA75	✓ (≈ 380 K)	×	✓[15]
IBP–PVA	✓ (≫ 600 K)	✓ (≫ 600 K)	n/a
IBP–PVPK12	×	×	×[12]
IMC–EUD	✓ (≈ 395 K)	✓ (≫ 600 K)	n/a
IMC–PLGA50	×	×	×[15]
IMC–PLGA75	×	×	×[15]
IMC–PVA	✓ (≫ 600 K)	✓ (≫ 600 K)	×[16]
IMC–PVPK12	×	×	×[17]
IMC–PVPVAc64	×	×	×[18]
NIF–PVPK12	×	–	×[12]
NIF–PVPK25	×	–	×[12]
NIF–PVPK30	×	–	×[12]
NIF–PVPVAc64	×	–	×[12]
NIF–SOL	×	–	×[12]
NPX–EUD	✓ (≈ 210 K)	×	n/a
NPX–PLGA50	×	×	×[15]
NPX–PLGA75	×	×	✓[15]
NPX–PVA	✓ (≫ 600 K)	✓ (≫ 600 K)	✓[16,b]
NPX–PVPK12	×	×	×[12]
NPX–PVPK25	×	×	×[12]
NPX–PVPK30	×	×	×[12]
NPX–PVPVAc64	✓ (≈ 265 K)	×	×[12]

(Continued on next page)

Table S3: Results of the Computational Analysis of AAPS Purely Predicted by PC-SAFT with Two Different API Parametrizations[a] *(Continued)*

System	REF	ALT	Exp. evidence of AAPS
	AAPS? (UCST)	AAPS? (UCST)	
NPX–SOL	×	×	×[12]
PCM–EUD	✓ ($\gg$ 600 K)	✓ ($\gg$ 600 K)	n/a
PCM–PLGA50	✓ ($\approx$ 285 K)	✓ ($\approx$ 305 K)	×[15]
PCM–PLGA75	✓ ($\approx$ 300 K)	✓ ($\approx$ 315 K)	×[15]
PCM–PVPK12	×	×	×[12]
PCM–PVPK30	×	×	×[19]
PCM–PVPVAc64	✓ ($\approx$ 445 K)	×	×[19]
SIM–PDL	✓ ($\approx$ 545 K)	–	n/a
SIM–PLGA50	✓ ($\approx$ 500 K)	–	n/a

[a] Acronyms and symbols: n/a = not available (AAPS analysis has not yet been performed); ✓= AAPS predicted/observed; × = AAPS not predicted/observed; – = not considered; T_g = glass-transition temperature; UCST = upper critical solution temperature (maximum AAPS temperature).

[b] Two T_g events were observed *via* differential scanning calorimetry for extrudates formulated via hot-melt extrusion with $w_{API, ini}$ = 30, 40, and 50 wt. % during a 12-month long-term physical stability study; $w_{API, ini}$ – initial API loading (on a mass basis).

References

(1) Kleiner, M.; Tumakaka, F.; Sadowski, G.; Latz, H.; Buback, M. Phase Equilibria in Polydisperse and Associating Copolymer Solutions: Poly(Ethene-Co-(Meth)Acrylic Acid)–Monomer Mixtures. *Fluid Phase Equilib.* **2006**, *241*, 113–123.

(2) Becker, F.; Buback, M.; Latz, H.; Sadowski, G.; Tumakaka, F. Cloud-Point Curves of Ethylene-(Meth)Acrylate Copolymers in Fluid Ethene up to High Pressures and Temperatures - Experimental Study and PC-SAFT Modeling. *Fluid Phase Equilib.* **2004**, *215*, 263–282.

(3) Lehmkemper, K.; Kyeremateng, S. O.; Heinzerling, O.; Degenhardt, M.; Sadowski, G. Impact of Polymer Type and Relative Humidity on the Long-Term Physical Stability of Amorphous Solid Dispersions. *Mol. Pharmaceutics* **2017**, *14*, 4374–4386.

(4) Cocchi, G.; De Angelis, M. G.; Sadowski, G.; Doghieri, F. Modelling Polylactide/Water/Dioxane Systems for TIPS Scaffold Fabrication. *Fluid Phase Equilib.* **2014**, *374*, 1–8.

(5) Prudic, A.; Lesniak, A. K.; Ji, Y. H.; Sadowski, G. Thermodynamic Phase Behaviour of Indomethacin/PLGA Formulations. *Eur. J. Pharm. Biopharm.* **2015**, *93*, 88–94.

(6) Peters, F. T.; Laube, F. S.; Sadowski, G. Development of a Group Contribution Method for Polymers within the PC-SAFT Model. *Fluid Phase Equilib.* **2012**, *324*, 70–79.

(7) Peters, F. T.; Herhut, M.; Sadowski, G. Extension of the PC-SAFT Based Group Contribution Method for Polymers to Aromatic, Oxygen- and Silicon-Based Polymers. *Fluid Phase Equilib.* **2013**, *339*, 89–104.

(8) Byun, H.-S.; Lee, B.-S. Liquid-Liquid Equilibrium of Hydrogen Bonding Polymer Solutions. *Polymer* **2017**, *121*, 1–8.

(9) Prudic, A.; Ji, Y.; Luebbert, C.; Sadowski, G. Influence of Humidity on the Phase Behavior of API/Polymer Formulations. *Eur. J. Pharm. Biopharm.* **2015**, *94*, 352–362.

(10) Tumakaka, F.; Gross, J.; Sadowski, G. Modeling of Polymer Phase Equilibria Using Perturbed-Chain SAFT. *Fluid Phase Equilib.* **2002**, *194-197*, 541–551.

(11) Sadowski, G. Technical University Dortmund, personal communication, 2021.

(12) Mathers, A. *Prediction of Drug Solubility in Polymer: Combined Experimental and Computational Study*; Unpublished doctoral dissertation, University of Chemistry and Technology, Prague, 2022.

(13) Mathers, A.; Hassouna, F.; Malinová, L.; Merna, J.; Růžička, K.; Fulem, M. Impact of Hot-Melt Extrusion Processing Conditions on Physicochemical Properties of Amorphous Solid Dispersions Containing Thermally Labile Acrylic Copolymer. *J. Pharm. Sci.* **2020**, *109*, 1008–1019.

(14) Iemtsev, A.; Hassouna, F.; Mathers, A.; Klajmon, M.; Dendisová, M.; Malinová, L.; Školáková, T.; Fulem, M. Physical Stability of Hydroxypropyl Methylcellulose-Based Amorphous Solid Dispersions: Experimental and Computational Study. *Int. J. Pharm.* **2020**, *589*, 119845.

(15) Iemtsev, A.; Hassouna, F.; Klajmon, M.; Mathers, A.; Fulem, M. Compatibility of Selected Active Pharmaceutical Ingredients with Poly(D, L-Lactide-Co-Glycolide): Computational and Experimental Study. *Eur. J. Pharm. Biopharm.* **2022**, *179*, 232–245.

(16) Mathers, A.; Pechar, M.; Hassouna, F.; Fulem, M. API Solubility in Semi-Crystalline Polymer: Kinetic and Thermodynamic Phase Behavior of PVA-Based Solid Dispersions. *Int. J. Pharm.* **2022**, *623*, 121855.

(17) Mathers, A.; Hassouna, F.; Klajmon, M.; Fulem, M. Comparative Study of DSC-Based Protocols for API-Polymer Solubility Determination. *Mol. Pharmaceutics* **2021**, *18*, 1742–1757.

(18) Iemtsev, A.; Zemánková, A.; Hassouna, F.; Mathers, A.; Klajmon, M.; Slámová, M.; Malinová, L.; Fulem, M. Ball Milling and Hot-Melt Extrusion of Indomethacin-L-Arginine-Vinylpyrrolidone-Vinyl Acetate Copolymer: Solid-State Properties and Dissolution Performance. *Int. J. Pharm.* **2022**, *613*, 121424.

(19) Mathers, A. *Various API–Polymer Solubility Datasets Determined via Differential Scanning Calorimetry*; Unpublished raw data, University of Chemsitry and Technology, Prague, 2022.
